# Copper and PFOS Co-Exposure Induces Synergistic Neurotoxicity via ROS-Mediated Mitophagy in *C. elegans*

**DOI:** 10.3390/toxics14060469

**Published:** 2026-05-27

**Authors:** Peixin Lu, Ying Zhang, Ruo Zhang, Kejia Liu, Wei Shi, Lu Lu, Qian Zhou, Yuepu Pu, Lihong Yin

**Affiliations:** Key Laboratory of Environmental Medicine Engineering, Ministry of Education of China, School of Public Health, Southeast University, 87 Dingjiaqiao Street, Nanjing 210009, China; 220234073@seu.edu.cn (P.L.); 101300315@seu.edu.cn (Y.Z.);

**Keywords:** Cu, PFOS, mitophagy, neurotoxicity, *C. elegans*

## Abstract

Copper (Cu) and perfluorooctanesulfonic acid (PFOS) are ubiquitous environmental pollutants that frequently co-occur, each capable of inducing neurotoxicity individually. However, the combined toxicity and interactive mechanisms of their co-exposure remain unclear, hindering an accurate assessment of their combined environmental health risks. Using the *Caenorhabditis elegans* model, we investigated the effects of co-exposure to environmentally relevant concentrations. Compared to individual exposures, co-exposure triggered synergistic neurotoxicity, characterized by the loss of dopaminergic (DAergic) and glutamatergic (GLUergic) neurons, aggravated locomotor deficits, massive accumulation of reactive oxygen species (ROS), and a severe decline in mitochondrial membrane potential, accompanied by substantial mitochondrial ultrastructural damage and accumulation of autophagosomes. Mechanistically, the excessive oxidative stress induced by co-exposure aberrantly and persistently activated the ROS-mediated mitophagy pathway, thereby impairing mitochondrial quality control. Critically, intervention with N-acetylcysteine (NAC), an antioxidant, effectively mitigated the co-exposure-induced deficits, identifying oxidative stress as the central driver of the synergistic toxicity. Our findings reveal a novel mechanism by which Cu and PFOS exert synergistic neurotoxicity via the oxidative-stress–mitophagy axis, providing key scientific evidence for refining the assessment of their combined environmental pollution risks.

## 1. Introduction

Copper (Cu), as an essential trace element, has become an increasingly prominent soil pollutant due to industrialization. Nationwide, 21.02% of agricultural soils exceed the Class II threshold (50 mg/kg) of the Soil Environmental Quality Standard [1,2], with the average concentration in south China reaching 61.71 mg/kg [1]. In aquatic ecosystems, sediment Cu concentrations in lakes like Chaohu generally exceed background values by 1.2–2.2 times [3]. Epidemiological studies have indicated that Cu accumulation in organisms is closely associated with neurodegenerative diseases (NDDs) such as Alzheimer’s disease (AD) and Parkinson’s disease (PD) [4,5]. The etiology and pathogenesis of most NDDs remain incompletely understood. Animal studies have demonstrated that elevated Cu levels induced ROS overproduction, disrupted mitochondrial metabolism, and impaired cognitive function by inhibiting mitophagy [6,7,8]. Our previous research has made progress in exploring the potential mechanisms underlying Cu-induced neurotoxicity. Persistent Cu accumulation in murine microglia reduces mitochondrial membrane potential (MMP) and decreases expression of PINK1 and Parkin, contributing to impaired mitophagy [9,10]. Perfluorooctane sulfonate (PFOS) is a representative persistent pollutant [11,12], high levels of which have been detected in key water bodies across China (the Yellow River reached 157.5 ng/L, exceeding the national standards by 3.9 times) [13]. A study showed that serum exposure levels in industrial areas (e.g., Hohhot: 34.42 ng/mL) reach 18 times those in low-exposure zones [14]. After entering the human body, PFOS accumulates in specific brain regions and disrupts neurotransmission, particularly in dopamine and glutamate systems, altering neurotransmitter levels, synaptic function, and neuronal protein expression [15,16]. This neurotoxicity involves dopamine system disruption and mitochondrial dysfunction. Zebrafish models demonstrate that PFOS reduces ATP production by 58%, induces ROS bursts, alters mitochondrial permeability, impairs mitochondrial function, affects apoptosis, and disrupts mitophagy [17,18,19,20,21].

Cu and PFOS, representative heavy metals and persistent organic pollutants [22], frequently co-occur in industrial areas, such as electronics manufacturing, electroplating, and firefighting foam sites. Natural releases and anthropogenic emissions (e.g., mining, agriculture) of Cu result in its widespread distribution in water, soil, and sediments [1,3,23]. PFOS, due to its chemical stability, can spread widely through industrial wastewater and firefighting foam leaks [11,12,24,25]. Both pollutants are ubiquitous and coexist long-term in the environment. Despite their shared neurotoxic mechanisms involving oxidative stress and mitochondrial damage, studies on their combined effects are scarce. The mechanisms underlying the co-exposure toxicity remain unclear. Therefore, this study established a *C. elegans* exposure model to investigate the mechanisms by which combined Cu–PFOS co-exposure disrupts the mitochondrial quality control network, providing new insights into the environmental etiology of NDDs.

## 2. Materials and Methods

### 2.1. Chemicals

Perfluorooctane sulfonic acid (PFOS, CAS: No. 1763-23-1) was purchased from Sigma-Aldrich (Burlington, MA, USA). Dimethyl sulfoxide (DMSO, Catalog No. 276855) was purchased from Sigma-Aldrich (Burlington, MA, USA). Copper (II) sulfate pentahydrate (CuSO_4_·5H_2_O, purity > 98%, CAS: No. 7758–99–8) was purchased from Sigma-Aldrich (Burlington, MA, USA). N-acetylcysteine (NAC), the reactive oxygen species (ROS) detection kit, and the TMRE mitochondrial membrane potential (MMP) detection kit were provided by Beyotime Biotechnology Co., Ltd. (Shanghai, China).

### 2.2. C. elegans Strains and Culture Methods

The wild-type N2 strain and mutant strains BR4006 [*pink-1*::GFP], CF1553 [*sod-3*::GFP], DA2123 [*lgg-1*::GFP], and MAH235 [*hlh-30*::GFP] were purchased from the Caenorhabditis Center (CGC, Minneapolis, MN 55455, USA). Following the protocol from Zhang [26], nematodes were cultured in Nematode Growth Medium (NGM) plates seeded with *Escherichia coli* (*E. coli*) OP50 at 20 °C. Each plate contained 200 µL of *E. coli* OP50 bacterial lawn (optical density at 570 nm = 2.0) [27]. Before exposure, gravid adults were washed three times with M9 buffer (3 g/L KH_2_PO_4_, 6 g/L Na_2_HPO_4_, 5 g/L NaCl) and lysed using a bleaching solution (50 μL/mL 5 M NaOH, 120 μL/mL NaClO) to collect eggs. Eggs were hatched in M9 buffer at 20 °C for 24 h to obtain synchronized L1 larvae, which were then transferred to 35 mm NGM plates pre-seeded with *E. coli* OP50 and containing 200 μL of the toxicant solution. After 48 h culture at 20 °C until reaching the young adult stage, worms were collected for subsequent analyses.

### 2.3. Cu and PFOS Exposure

According to the Surface Water Environmental Quality Standard [28], the Cu^2+^ limit for Class I water bodies is 0.01 mg/L. Accordingly, the Cu^2+^ concentration employed in this study was 10 μg/L, prepared by diluting a stock solution (1 g/L). According to the Chinese Drinking Water Quality Standard [29], PFOS in drinking water should not exceed 40 ng/L. Thus, the PFOS concentration in this study was set at 40 ng/L.

All toxicant solutions were prepared in K-medium. Synchronized L1 worms were exposed to 0 (K-medium as control), 10 µg/L Cu^2+^, 40 ng/L PFOS, and 10 µg/L Cu^2+^ + 40 ng/L PFOS co-exposure until the young adult stage (approximately 48 h), then assessed for locomotor behavior, neuronal morphology, oxidative stress, mitochondrial function, and gene expression. Experiments were performed in triplicate.

### 2.4. Locomotor Behavior Assay

Head thrashes, body bends, pharyngeal pump rates, and defecation intervals were used as locomotor behavior indicators. After 48 h exposure, twenty worms were scored for head thrashes and body bends over 30s in M9 buffer. Another 20 worms were transferred to OP50-seeded plates for pumping frequency and defecation interval measurements.

### 2.5. ROS Level Detection

ROS levels were measured by H_2_DCFDA probe (Beyotime, Nanjing, China). After 48 h exposure, worms were incubated in 1 mL of 10 μM H_2_DCFDA in the dark for 2 h (with a positive control), then washed, anesthetized with 2 mM levamisole, and mounted. At least 20 worms were photographed under a Zeiss AX10 microscope (40×, Jena, Germany) and analyzed with ImageJ1.53 software.

### 2.6. Tetramethylrhodamine Ethyl Ester Perchlorate Salt (TMRE) Staining

MMP was assessed by TMRE staining. After 48 h exposure, worms were incubated with 4 μM TMRE for 90 min in the dark (with a positive control), washed, anesthetized with levamisole, and mounted. At least 20 worms were photographed under a Zeiss microscope (10×) and analyzed with ImageJ 1.53 software.

### 2.7. Transmission Electron Microscopy (TEM)

After 48 h of exposure, nematodes were washed three times with M9 buffer and fixed overnight at 4 °C in 2.5% glutaraldehyde. Subsequent processing, including post-fixation with 1% osmium tetroxide, gradient ethanol dehydration, resin embedding, ultrathin sectioning (70–100 nm), and staining with uranyl acetate and lead citrate, was performed by Wuhan Servicebio Technology Co., Ltd. (Wuhan, Hubei, China). Sections were imaged using a Hitachi transmission electron microscope (Tokyo, Japan) at an accelerating voltage of 80.0 kV.

### 2.8. Lysosomal Staining with LysoTracker Green

Lysosomal staining was performed using LysoTracker Green (Beyotime, Shanghai, China). After 48 h of treatment, worms were washed with M9 buffer and subsequently incubated in the dark for 90 min with the probe (1:2000 in M9 buffer), then washed, anesthetized with levamisole, and mounted. At least 20 worms were photographed under a Zeiss microscope (10×) and analyzed with ImageJ software.

### 2.9. Quantitative Real-Time Polymerase Chain Reaction (qRT-PCR)

Total RNA was extracted using RNeasy separation reagents and reverse-transcribed into cDNA with the Prime Script RT kit. qRT-PCR was performed on a StepOnePlus system (Applied Biosystems, Foster City, CA, USA) using the SYBR Premix Ex Taq™, with cycling conditions following the manufacturer’s protocol. Actin served as the housekeeping gene, and the relative expression levels were calculated using the 2^−ΔΔCT^ method with three technical replicates. Primer sequences are listed in Supplementary Table S1.

### 2.10. NAC Assay

N-acetylcysteine (NAC), a thiol-containing antioxidant, was used to rescue oxidative damage in L1-stage N2 worms exposed to 0 (K-medium as control), 10 µg/L Cu^2+^, 40 ng/L PFOS, and 10 µg/L Cu^2+^ + 40 ng/L PFOS co-exposure, with or without 1 mM NAC. After 48 h, we assessed head thrash frequency, defecation cycle, ROS levels, MMP (TMRE staining), and gene expression levels (qRT-PCR).

### 2.11. Data Analysis

We analyzed the data using GraphPad Prism 7 and presented the mean ± SD from three independent experiments. Differences among multiple groups were analyzed using one-way ANOVA, followed by Dunnett’s test for comparisons with or without NAC treatment, with Bonferroni correction for multiple comparisons.

## 3. Results

### 3.1. Effects of Cu and PFOS Co-Exposure on Nervous System and Locomotor Behavior in C. elegans

**Figure 1:**
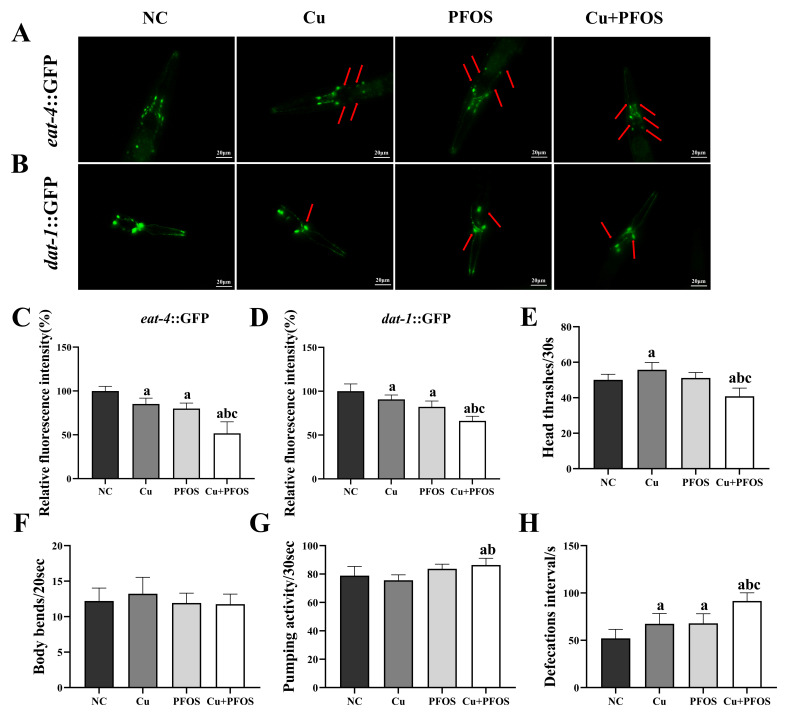
Exposure to Cu- and PFOS-caused neurotoxicity in *C. elegans*. (**A**) Representative images of *eat-4*::GFP, scale bar = 20 μm. (**B**) Representative images of *dat-1*::GFP, scale bar = 20 μm. (**C**) Relative fluorescence intensity of *eat-4*::GFP. (**D**) Relative fluorescence intensity of *eat-4*::GFP. (**E**) Head thrashes. (**F**) Body bends. (**G**) Pumping activity. (**H**) Defecation interval. The red arrows indicate the lost or diminished neurons. Compared with the control group, a, *p* < 0.05; compared with the Cu group, b, *p* < 0.05; compared with the PFOS group, c, *p* < 0.05.

Studies have shown that both Cu and PFOS can disrupt the nervous system upon human bioaccumulation, particularly affecting dopaminergic (DAergic) and glutamatergic (GLUergic) systems [30]. Locomotor behavior in *C. elegans* serves as a representative quantitative indicator of neurotoxicity [31]. After 48 h exposure from the L1 stage to adulthood, we assessed neuronal damage and locomotor behavior. Results showed that compared to controls, worms individually exposed to Cu or PFOS significantly reduced fluorescence intensity in *dat-1*::GFP and *eat-4*::GFP reporter strains, accompanied by partial neuronal loss and synaptic damage (Figure 1A,B). These effects were more severe in the co-exposure group, with further significant fluorescence reduction (Figure 1C,D). Cu and PFOS exposure individually increased head thrash frequency by 11.33% and 2.15%, respectively, while co-exposure significantly depressed it by 18.46%. Defecation cycle was prolonged by 29.58% (Cu) and 30.64% (PFOS) individually, but co-exposure caused a 75.96% prolongation. Body bending and pharyngeal pumping were minimally affected (Figure 1E–H). These findings demonstrate that Cu and PFOS exposure caused ‌DAergic and GLUergic neuron damage and locomotor deficits in *C. elegans*, with co-exposure producing stronger effects, suggesting a synergistic interaction between Cu and PFOS.

### 3.2. Effects of Cu and PFOS Co-Exposure on Oxidative Stress Levels in C. elegans

**Figure 2:**
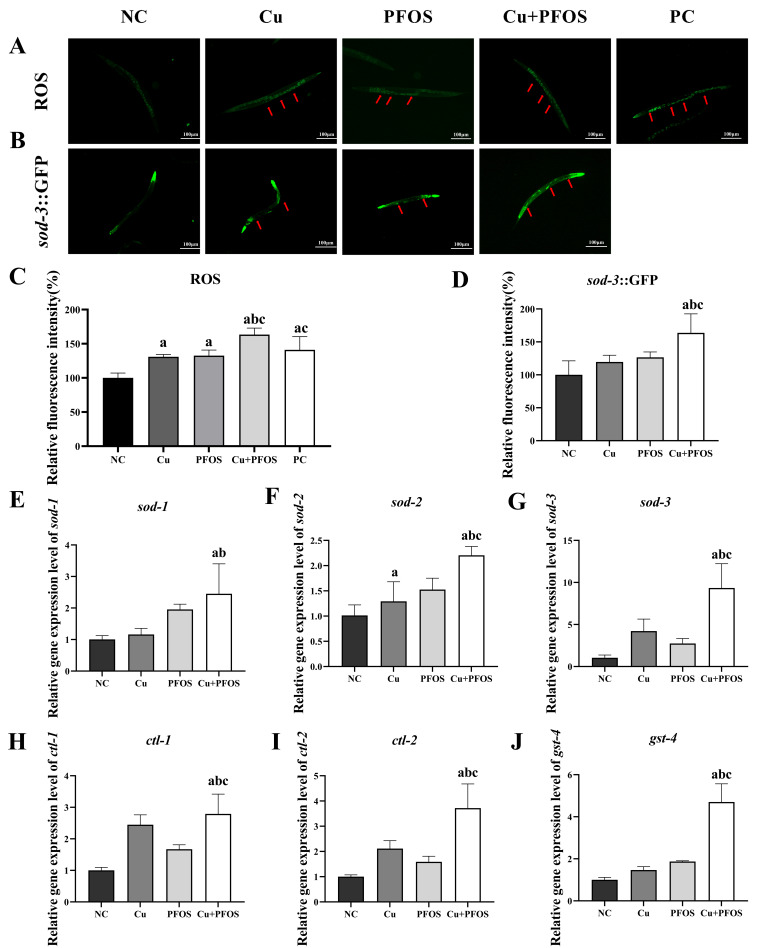
Exposure to Cu- and PFOS-induced oxidative stress in *C. elegans*. (**A**) Representative images of H2DCFDA probe staining, with red arrows indicating the ROS-accumulated regions, scale bar = 100 μm. (**B**) Representative images of *sod-3:*:GFP, with red arrows highlighting the regions of enhanced fluorescence, scale bar = 100 μm. (**C**) Quantitative analysis of relative H2DCFDA fluorescence intensity. (**D**) Relative fluorescence intensity of *sod-3*::GFP. (**E**) Relative expression levels of *sod-1*. (**F**) Relative expression levels of *sod-2*. (**G**) Relative expression levels of *sod-3*. (**H**) Relative expression levels of *ctl-1*. (**I**) Relative expression levels of *ctl-2*. (**J**) Relative expression of *gst-4*. PC: positive control; compared with the control group, a, *p* < 0.05; compared with the Cu group, b, *p* < 0.05; compared with the PFOS group, c, *p* < 0.05.

Intestinal ROS levels were detected using the H_2_DCFDA probe. Results showed that co-exposure resulted in significantly higher ROS levels than individual exposures, indicating a synergistic effect of Cu and PFOS in inducing oxidative stress in *C. elegans* (Figure 2A,C). In the *sod-3*::GFP reporter strain, *sod-3* protein and GFP can be observed as green fluorescence in the head, tail, and surrounding vulval areas under fluorescence microscopy. Fluorescence intensity in the co-exposure group was markedly increased compared to individual exposure, reflecting elevated *sod-3* expression (Figure 2B,D). Further, qRT-PCR analysis revealed that co-exposure significantly upregulated the expression of antioxidant genes *sod-1*, *sod-2*, *sod-3*, *ctl-1*, *ctl-2*, and *gst-4* compared to controls (*p* < 0.05, Figure 2E–J). Collectively, these results demonstrated that co-exposure to Cu and PFOS induced a stronger increase in oxidative stress levels than individual exposures, leading to an imbalance in the antioxidant system.

### 3.3. Effects of Cu and PFOS Co-Exposure on Mitochondria Damage in C. elegans

**Figure 3:**
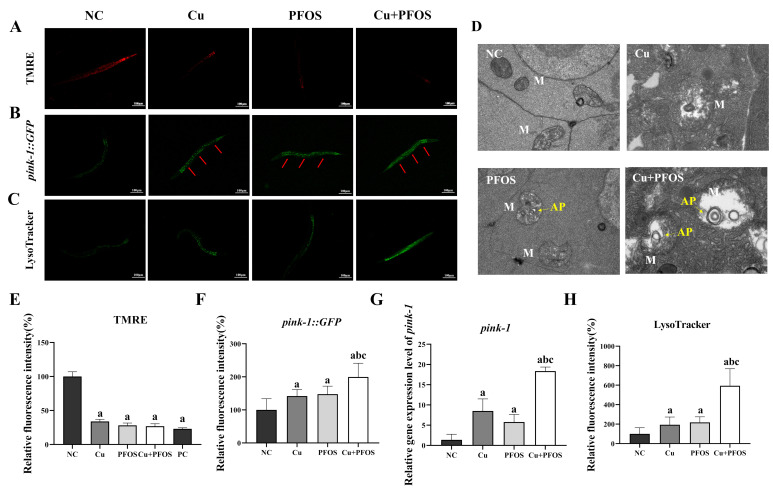
Exposure to Cu- and PFOS-induced mitochondrial damage. (**A**) Representative TMRE staining images, scale bar = 100 μm. (**B**) Representative fluorescence image of *pink-1:*:GFP, with red arrows highlighting the increased fluorescence, scale bar = 100 µm. (**C**) Representative fluorescence image of LysoTracker staining, scale bar = 100 µm. (**D**) Representative TEM images of mitochondria, scale bar = 500 nm. M: mitochondria, AP: autophagosomes. (**E**) Relative fluorescence intensity of TMRE probe staining. (**F**) Relative fluorescence intensity of *pink-1*::GFP. (**G**) Relative expression levels of *pink-1*. (**H**) Relative fluorescence intensity of LysoTracker staining. Compared with the control group, a, *p* < 0.05; compared with the Cu group, b, *p* < 0.05; compared with the PFOS group, c, *p* < 0.05.

Mitochondrial dysfunction is typically accompanied by loss of MMP. Disruption of MMP is a primary indicator of mitochondrial dysfunction [32]. Decreased MMP impairs mitochondrial electron transport chain function, reduces metabolic oxygen consumption, and depletes ATP [33]. To assess mitochondrial damage, MMP was measured using TMRE staining. TMRE, a cell-permeable cationic fluorescent dye sensitive to MMP, accumulates in healthy mitochondria and emits bright orange-red fluorescence. During apoptosis, mitochondrial fluorescence intensity significantly decreases. Results (Figure 3A,E) showed that compared to the control group, MMP was significantly reduced in worms individually exposed to Cu or PFOS. Co-exposure caused more severe MMP reduction, comparable to the positive control.

This study utilized the *pink-1*::GFP transgenic strain to assess PINK1 protein expression. The results (Figure 3B,F) revealed that *pink-1* expression was significantly increased in both the Cu and PFOS individual exposure groups, with a more pronounced upregulation in the co-exposure group.

LysoTracker Green is an acidotropic probe that selectively accumulates in acidic compartments, reflecting lysomal abundance and acidity [34]. The results showed that fluorescence intensity was significantly increased in all exposure groups compared to the control, with the greatest enhancement in the co-exposure group (Figure 3C,H). This indicates elevated lysosomal number and/or acidification following exposure.

Transmission electron microscopy (TEM) revealed mitochondrial ultrastructure. Mitochondria in the control group exhibited regular spherical or elongated shapes with distinct cristae. Individual exposure to Cu or PFOS induced mild mitochondrial swelling, partial membrane damage, slight cristae disruption, and minor autophagosome formation. Co-exposure induced extensive mitochondrial vacuolation, marked mitochondrial swelling, disrupted cristae, severe structural damage, and accumulation of autophagosomes (Figure 3D).

### 3.4. Effects of Co-Exposure to Cu and PFOS on Autophagy in C. elegans

**Figure 4:**
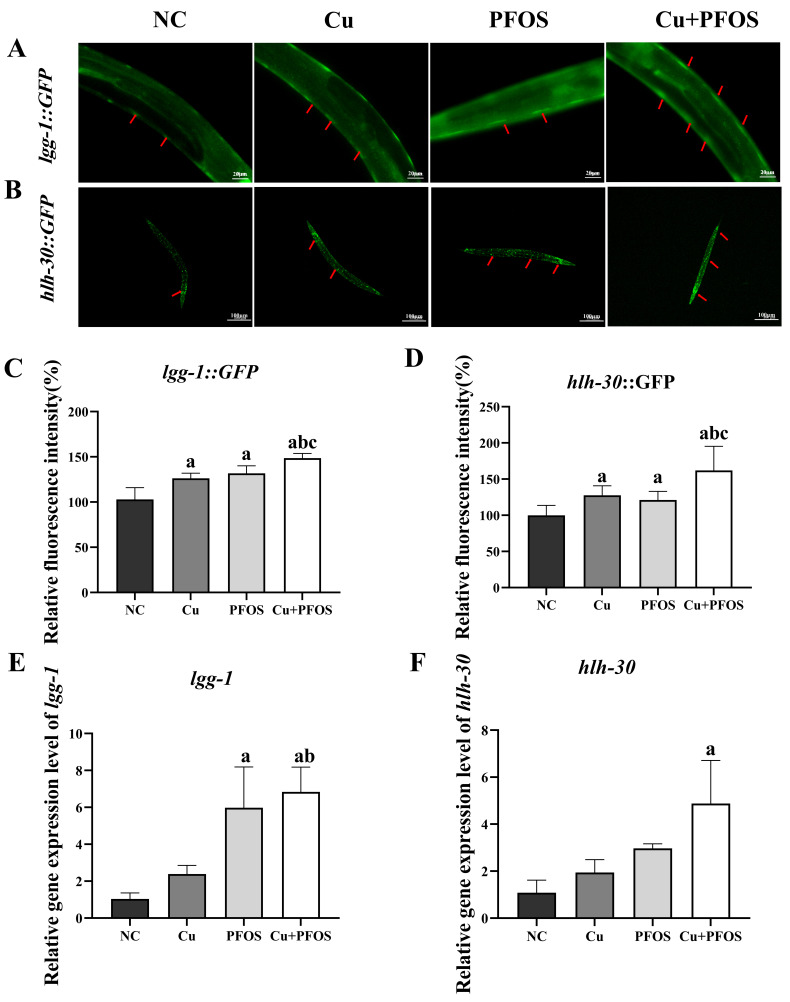
Exposure to Cu- and PFOS-induced mitophagy in *C. elegans*. (**A**) Representative fluorescence image of *lgg-1*::GFP, with red arrows highlighting the fluorescent puncta of LGG-1, scale bar = 20 µm. (**B**) Representative fluorescence image of *hlh-30:*:GFP, with red arrows highlighting the hlh-30::GFP fluorescent aggregates, scale bar = 100 µm. (**C**) Relative fluorescence intensity of *lgg-1*::GFP. (**D**) Relative fluorescence intensity of *hlh-30:*:GFP. (**E**) Relative expression levels of *lgg-1*. (**F**) Relative expression levels of *hlh-30*. Compared with the control group, a, *p* < 0.05; compared with the Cu group, b, *p* < 0.05; compared with the PFOS group, c, *p* < 0.05.

The human ubiquitin-like protein LC3 is essential for autophagosome formation [35]. Using the *lgg-1*::GFP reporter strain, the puncta observed under fluorescence microscopy serve as markers for autophagosomes. Results showed an increase in the number of fluorescent puncta in Cu- or PFOS-exposed worms, while the co-exposure group showed more bright spots (Figure 4A,C).

HLH-30, the *C. elegans* ortholog of the mammalian transcription factor EB (TFEB), regulates autophagy and lysosomal biogenesis by promoting lysosomal gene expression, thereby supporting PINK1/Parkin-mediated mitophagy. Stress conditions, such as oxidative stress, can trigger the nuclear translocation of *hlh-30*, a process directly observable in the *hlh-30*::GFP reporter strain [36]. Using *hlh-30*::GFP, the fluorescence intensity was significantly increased following individual exposure, with a more pronounced upregulation in the co-exposure group (Figure 4B,D), consistent with the observed increase in lysosomal number. qRT-PCR further confirmed that compared to the control group, the mRNA expression of *hlh-30* and *lgg-1* was significantly upregulated by Cu or PFOS, with more pronounced changes in the co-exposure group (Figure 4E,F).

### 3.5. NAC Reversed Locomotor Deficits and Neuronal Damage Induced by Cu–PFOS Co-Exposure

**Figure 5:**
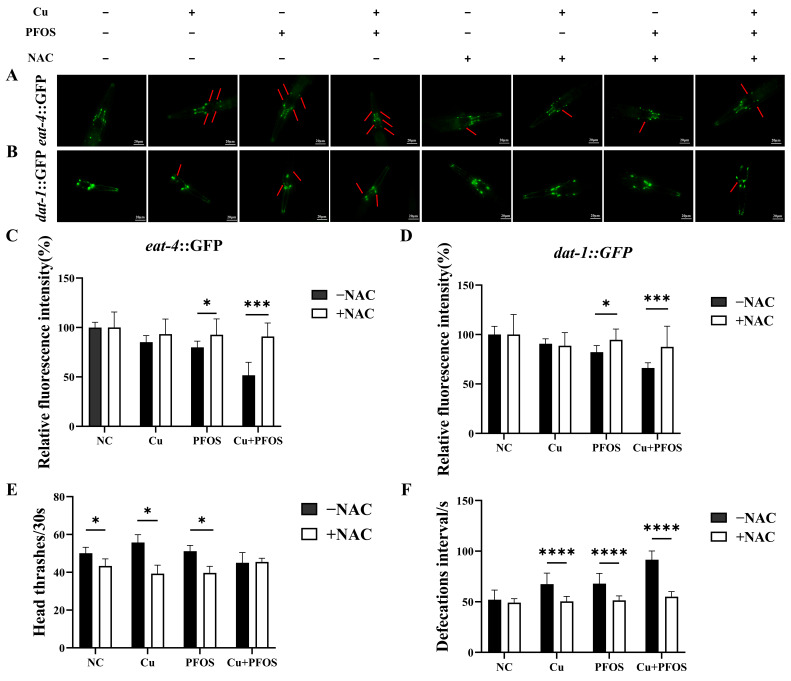
Effects of NAC treatment on neuronal damage and locomotor deficits induced by co-exposure to Cu and PFOS. (**A**) Representative fluorescence image of *eat-4*::GFP, scale bar = 100 µm. (**B**) Representative fluorescence image of *dat-1*::GFP, scale bar = 100 µm. (**C**) Relative fluorescence intensity of *eat-4*::GFP. (**D**) Relative fluorescence intensity of *dat-1*::GFP. (**E**) Head thrashes. (**F**) Defecation intervals. The red arrows indicate the lost or diminished neurons. * *p* < 0.05, *** *p* < 0.001, **** *p* < 0.0001.

Oxidative stress is a prerequisite for mitophagy [37]. As documented, sustained oxidative damage triggers mitochondrial ROS (mtROS) burst, leading to PINK1 accumulation, Parkin-dependent autophagy activation, and ultimately neuronal deficits [38]. We employed NAC to investigate the role of oxidative stress in Cu- and PFOS-induced mitophagy. The results demonstrated that NAC treatment partially reversed the neuronal deficits, as evidenced by the recovered fluorescence intensity in *eat-4*::GFP and *dat-1*::GFP reporter strains (Figure 5A–D), accompanied by reduced neuronal loss and synaptic disruption. Furthermore, head thrash frequency and defecation intervals were restored to control levels, with no statistically significant difference (Figure 5E,F). These data indicate that the NAC alleviated the neurotoxicity and locomotor deficits induced by co-exposure, supporting oxidative stress as a primary driver of the observed toxicity.

### 3.6. NAC Reverses Oxidative Damage Induced by Co-Exposure to Cu and PFOS

**Figure 6:**
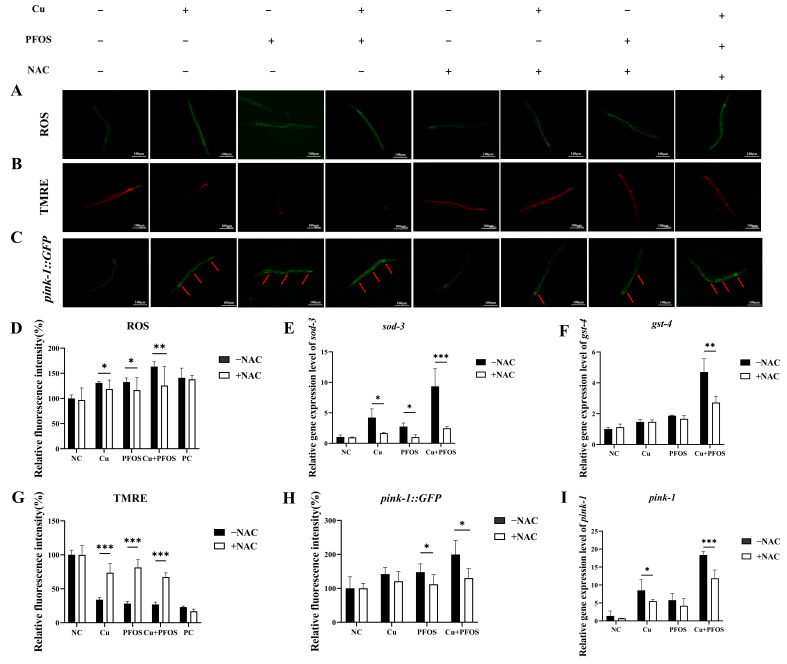
Effects of NAC treatment on oxidative damage induced by co-exposure to Cu and PFOS. (**A**) Representative fluorescence image of H2DCFDA staining, scale bar = 100 µm. (**B**) Representative fluorescence image of TMRE staining, scale bar = 100 µm. (**C**) Representative fluorescence image of *pink-1*::GFP, with red arrows highlighting the increased fluorescence, scale bar = 100 µm. (**D**) Relative fluorescence intensity of H2DCFDA. (**E**) Relative expression level of *sod-3*. (**F**) Relative expression level of *gst-4*. (**G**) Relative fluorescence intensity of TMRE. (**H**) Relative fluorescence intensity of *pink-1*::GFP. (I) Relative expression level of *pink-1*. * *p* < 0.05, ** *p* < 0.01, *** *p* < 0.001.

NAC treatment reversed the exposure-induced elevation in ROS levels (Figure 6A,D) and loss of MMP (Figure 6B,G), restoring both to near-control levels. Based on preliminary qRT-PCR screening, *sod-3* and *gst-4*, which showed the most pronounced response to Cu and PFOS co-exposure, were selected as core biomarkers of oxidative stress for validation. qRT-PCR analysis after NAC treatment showed that expression levels of *sod-3* and *gst-4* were significantly reduced, with *sod-3* returning to control levels, indicating effective alleviation of oxidative stress levels (Figure 6E,F). NAC treatment also reduced *pink-1*::GFP fluorescence and *pink-1* mRNA expression to near-control levels, confirming remediation of MMP impairment (Figure 6C,H,I). Collectively, these results indicate that NAC intervention alleviates oxidative stress and rescues mitochondrial dysfunction caused by Cu and PFOS co-exposure.

### 3.7. NAC Reverses Mitophagy Disruption Induced by Exposure to Cu and PFOS

**Figure 7:**
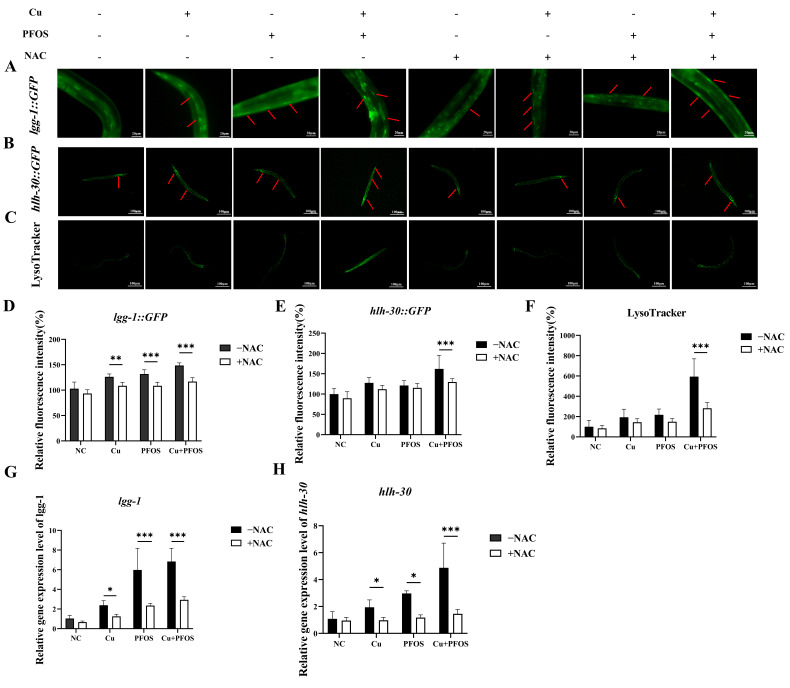
Effects of NAC treatment on autophagy function. (**A**) Representative fluorescence image of *lgg-1*::GFP, with red arrows highlighting the fluorescent puncta of LGG-1, scale bar = 20 µm. (**B**) Representative fluorescence image of *hlh-30:*:GFP, with red arrows highlighting the hlh-30::GFP fluorescent aggregates, scale bar = 100 µm. (**C**) Representative fluorescence image of LysoTracker staining, scale bar = 100 µm. (**D**) Relative fluorescence intensity of *lgg-1*::GFP. (**E**) Relative fluorescence intensity of *hlh-30:*:GFP. (**F**) Relative fluorescence intensity of LysoTracker staining. (**G**) Relative expression level of *lgg-1*. (**H**) Relative expression level of *hlh-30*. * *p* < 0.05, ** *p* < 0.01, *** *p* < 0.001.

NAC treatment on the reporter strains *lgg-1*::GFP and *hlh-30*::GFP reduced both the number of fluorescent puncta and relative fluorescence intensity, showing no significant difference from the controls (Figure 7A,B,D,E). LysoTracker staining showed a significant decrease in lysosome number following NAC treatment (Figure 7C,F), indicating decreased autophagic activity. Concurrently, the expression of *lgg-1* and *hlh-30* was significantly downregulated after NAC administration (Figure 7G–I). Based on the downregulation of both *pink-1*::GFP fluorescence and *pink-1* mRNA expression described in Section 3.6, we conclude that mitophagy was reduced after NAC treatment. In addition, the antioxidant effectively reversed the excessive mitophagy induced by oxidative stress.

## 4. Discussion

Environmental mixtures of pollutants can interact through additive, antagonistic, or synergistic effects [8,39]. Cu and PFOS are frequently detected simultaneously in industrial areas and contaminated water bodies [40,41], and growing evidence points to their close geochemical and biological association. In natural waters, Cu^2+^ enhances PFOS adsorption through ternary complexation, prompting their co-occurrence [42]. Filed studies have further revealed significant positive correlations between Cu and PFAS levels in the livers of top marine predators, reflecting their intertwined environmental fate [43]. A spatiotemporal analysis of surface- and groundwater have identified PFOS and Cu within the same contaminant clusters [44]. These findings indicate that Cu and PFOS not only coexist but may also influence each other’s transport and bioavailability. Existing studies indicate that Cu and PFOS converge in several shared neurotoxicity pathways. Cu overload induces cognitive dysfunction by oxidative stress, excessive ROS generation, mitochondrial disruption, and impaired mitophagy [6,7,45], while PFOS accumulation in the human brain can damage the nervous system, causing dysregulation of the dopamine system and mitochondrial dysfunction. Despite their distinct chemical properties, they share common neurotoxic mechanisms involving oxidative stress, mitochondrial dysfunction, and dopaminergic system impairment. Emerging evidence indicates that co-exposure to heavy metals and organic compounds sharing common toxic mechanisms frequently produces synergistic effects. For instance, co-exposure to manganese and methylmercury induces dopaminergic neurotoxicity and mitochondrial dysfunction in *C. elegans* [46]. Similarly, co-exposure to cadmium and polystyrene nanoplastics exhibits synergistic neurotoxicity [47], and co-exposure to cadmium and fumonisin B1 exacerbates oxidative damage, mitochondrial dysfunction, and ferroptosis [48]. A recent study by Niu et al. (2025) further demonstrated that co-exposure to polystyrene nanoplastics and cadmium synergistically aggravated oxidative stress, mitochondrial membrane depolarization, ATP depletion, and dopaminergic neuronal damage in *C. elegans*, effects that were mitigated by the antioxidant NAC [47]. These findings closely parallel the synergistic pattern observed for Cu and PFOS in the present study, reinforcing the notion that pollutant pairs sharing oxidative stress and mitochondrial dysfunction pathways tend to produce synergistic neurotoxicity. Additionally, polystyrene microplastics have been shown to potentiate thallium-induced neurotoxicity in *C. elegans*, disrupting multiple neurotransmitter systems under co-exposure conditions [49]. We therefore hypothesized that the Cu and PFOS co-exposure might produce synergistic neurotoxicity.

To systematically assess their combined toxicity, this study exposed *C. elegans* to environmentally relevant concentrations of Cu and PFOS individually and in combination. Co-exposure to Cu and PFOS induced more severe locomotor deficits than individual exposures, suggesting enhanced neurotoxicity. Molecular-level findings were consistent with the phenotypic observations: co-exposure induced significantly greater oxidative stress and mitophagic dysfunction than individual exposures.

Based on various evidence from the literature [8,46,47,48], we propose that environmentally relevant concentrations of Cu and PFOS likely act synergistically to induce sustained PINK1/Parkin-mediated mitophagy via oxidative stress. The consequent excessive autophagosome accumulation and impaired degradation drive neurotoxicity.

Upon environmental stress, *C. elegans* activates key antioxidant genes [50]. The superoxide dismutase (SOD) family constitutes the primary defense against ROS by catalyzing the dismutation of superoxide anions (O^2−^) into hydrogen peroxide (H_2_O_2_) and molecular oxygen. The resulting H_2_O_2_ is subsequently detoxified by catalase (CAT) and glutathione peroxidase (GPx) [51,52]. Specifically, *sod-1* is ubiquitously expressed in the cytoplasm across nearly all tissues [53]. *sod-2* primarily localizes to mitochondria and scavenges the superoxide anion (O_2_^−^) generated by the electron transport chain. *sod-3*, a cytosolic isoform, catalyzes the dismutation of superoxide into hydrogen peroxide, halting ROS propagation. It is a classic oxidative stress biomarker [54]. The catalase (CTL) family degrades hydrogen peroxide (H_2_O_2_). In this study, we focused on the cytosolic isoforms *ctl-1* and *ctl-2*, as mitochondrial H_2_O_2_ in *C. elegans* is primarily cleared via the thioredoxin system (e.g., *trx-1*), making *ctl-3* functionally minor and therefore excluded from our investigation. *gst-4* detoxifies oxidative damage products, promotes clearance of oxidatively damaged proteins via autophagy, and conjugates glutathione to toxic substrates, thereby eliminating excess intracellular ROS and xenobiotics. These actions collectively contribute to neuronal protection and lifespan extension [54]. Based on this framework, we selected *sod-1*, *sod-2*, *sod-3*, *ctl-1*, *ctl-2*, and *gst-4* for investigation.

Existing research indicates that co-exposure to microplastics and Cr induces synergistic neurotoxicity in *C. elegans* [47]. Studies on aquatic organisms, however, suggest that PFOS combined with other environmental stressors typically exhibits an additive effect. In contrast, our results demonstrate that co-exposure to Cu and PFOS induced the highest ROS levels and strongest *sod-3*::GFP fluorescence, accompanied by a pronounced decline in MMP and marked upregulation of antioxidant genes. These findings indicate that co-exposure to Cu and PFOS substantially exacerbates oxidative stress and promotes MMP collapse in *C. elegans*.

Excessive ROS accumulation impairs mitochondrial structure and function, disrupting cellular homeostasis [6,55]. In response, mitochondria engage mitophagy to regulate ROS levels. This establishes an essential regulatory circuit, as mitochondria both generate ROS through oxidative phosphorylation and harbor mechanisms for its clearance. Consequently, the maintenance of cellular homeostasis relies on a critical balance between these opposing mitochondria functions.

The PINK1/Parkin pathway serves as a core mitophagy mechanism, and its dysfunction is implicated in major diseases such as Parkinson’s disease [56,57]. PINK1 (ortholog of *pink-1* in *C. elegans*) is a mitochondria-targeted kinase that acts as a sensor of mitochondrial damage. Upon loss of membrane potential, PINK1 accumulates on the outer mitochondrial membrane (OMM), recruiting and phosphorylating the E3 ubiquitin ligase Parkin (ortholog of *pdr-1* in *C. elegans*) to initiate autophagy. Activated Parkin ubiquitinates OMM proteins (e.g., VDAC, Miro), subsequently recruiting autophagy receptor proteins that bind to LC3-II (ortholog of *lgg-1* in *C. elegans*). This leads to the encapsulation of damaged mitochondria and lysosomal degradation. Concurrently, Parkin activates the kinase TBK1, which in turn phosphorylates the transcription factor TFEB (ortholog of *C. elegans: hlh-30*), enhancing autophagosome formation and lysosomal clearance capacity [48,56,57,58]. Based on this pathway, we selected key PINK1/Parkin-related genes in *C. elegans*—*pink-1*, *hlh-30*, and *lgg-1*—for investigation. Co-exposure induced sustained upregulation of these mitophagy genes, accompanied by increased fluorescence in corresponding reporter strains. TEM further revealed substantial mitochondrial structural damage and autophagosome formation. Under physiological conditions, this pathway is appropriately activated to remove damaged mitochondria [59]. However, the sustained and widespread activation observed here, particularly autophagosomes accumulation, suggests impaired progression of the degradative phase. This dysregulation of mitophagy flux not only fails to achieve protective clearance but may instead exacerbate metabolic and homeostatic crises, a pathology reminiscent of mitochondrial quality control in NDDs [60].

To further analyze the mitochondrial dysfunction, we used the antioxidant NAC for a rescue experiment. NAC is widely used in *C. elegans* and exhibits a broad effective concentration range. Multiple studies have shown that NAC exerts significant antioxidant and neuroprotective effects at concentrations between 0.5 mM and 10 mM [61,62,63,64]. However, its dose–response relationship follows an inverted U-shaped curve [65], where too low a concentration is ineffective and too high a concentration becomes toxic. Gonzales-Moreno et al. (2023) reported that 0.5 mM NAC effectively alleviates acute oxidative damage in nematodes, whereas concentrations of 2.5 mM and above significantly increase nematode mortality in a dose-dependent manner [62]. Furthermore, studies have shown that 5 mM-and-higher concentrations of NAC shorten the lifespan of *C. elegans* [66]. Therefore, 1 mM was selected as the working concentration of NAC in this study. NAC treatment significantly restored ROS levels, mitigated mitochondrial damage, normalized autophagic activity, and alleviated neurotoxicity. The protective effect of NAC against combined pollutant-induced neurotoxicity is consistent with the findings of Niu et al. (2025), who reported that NAC pretreatment mitigated oxidative stress and synaptic damage in a nanoplastics–cadmium co-exposure model, further supporting oxidative stress as an upstream driver of the synergistic mitochondrial impairment observed across different pollutant combinations [47]. These results demonstrate that oxidative stress drives mitophagy disruption underlying the synergistic neurotoxicity of Cu and PFOS. Consequently, NAC shows potential for reversing Cu- and PFOS-induced neurotoxicity, providing a prospective intervention target for preventing neuronal damage from their co-exposure.

## 5. Conclusions

In summary, co-exposure to environmental concentrations of Cu and PFOS enhanced oxidative stress in *C. elegans*, leading to persistent ROS accumulation, oxidative damage, mitochondrial structural injury, and reduced MMP. The PINK1/Parkin-mediated mitophagy was persistently activated, as evidenced by elevated expression levels of *pink-1*, *hlh-30*, and *lgg-1*. This disruption of neuronal energy metabolism and cellular homeostasis ultimately induced neurotoxic effects including early-onset locomotor deficits. These findings provide new insights into the toxic mechanisms of co-exposure to Cu and PFOS and may also offer valuable information for assessing the risks of co-exposure to other heavy metals and PFOS in natural environments.

## Data Availability

The raw data supporting the conclusions of this article will be made available by the authors on request.

## Supplementary Materials

The following supporting information can be downloaded at: https://www.mdpi.com/article/10.3390/toxics14060469/s1, Table S1 . The gene primer used in the study.
