# Supplementary material for: Copper and PFOS Co-Exposure Induces Synergistic Neurotoxicity via ROS-Mediated Mitophagy in C. elegans
Source: Toxics. 2026 May 27;14(6):469. doi: 10.3390/toxics14060469 (PMC13306770; doi:10.3390/toxics14060469)
Supplement: Supplementary file 1 [file toxics-14-00469-s001.zip › toxics-4298801-supplementary.pdf]

Supplementary Materials for

Copper and PFOS Co-exposure induce synergistic neurotoxicity via ROS-mediated mitophagy in *C. elegans*

Peixin Lu[#], Ying Zhang[#], Ruo Zhang, Kejia Liu, Wei Shi, Lu Lu, Qian Zhou, Yuepu Pu, Lihong Yin*

Key Laboratory of Environmental Medicine Engineering, Ministry of Education of China; School of Public Health, Southeast University, Nanjing210009, Jiangsu, China

This file includes

Table S1

Table S1

Table S1. The gene primer used in the study

Gene Name	Forward（5'-3'）	Reverse（5'-3'）
ctl-1	GTCGTTCATGCCAAGGGAG	ACGAACGAGAAGTGGTGTCT
ctl-2	TCAACCCCGTCAATTCTGGG	TGAGAGCGAGCCTGTTTCTG
gst-4	CCAGCCCGTGATGATTTCT	GCAATCACAATATCAGCCCAAG
hlh-30	CCCAACAGCAGCAGAAATCG	GAAGCCGGATGAGGACACAA
lgg-1	TACCAGGACCATCACGAGGA	AGGGGCTTTGGGTTTCCATT
pdr-1	CGGCAAACTGCGGGTTTATT	TCACAAACACAGCACTCGGT
pink-1	GAGGAAATGCCAAGACAAAAGCACC	AGGCCTCCAGCTGCCCATGT
sod-1	GTGATTCCACCAACGGATGC	TTGTCCGGCATGAACAACCA
sod-2	GCTCTTCAGCCAGCTCTCAA	CCAGAGATCCGAAGTCGCTC
sod-3	AGCATCATGCCACCTACGTGA	CACCACCATTGAATTTCAGCG
β-actin	CAAAATGTGTGACGACGAGG	ATGACTCCTTGATGACGTGG
